# Potential Neuroprotective Effect of Melatonin in the Hippocampus of Male BTBR Mice

**DOI:** 10.3390/nu16111652

**Published:** 2024-05-28

**Authors:** Matteo Bonetti, Lorena Giugno, Elisa Borsani, Francesca Bonomini

**Affiliations:** 1 Division of Anatomy and Physiopathology, Department of Clinical and Experimental Sciences, University of Brescia, 25123 Brescia, Italy; matteo.bonetti@unibs.it (M.B.); lorena.giugno@unibs.it (L.G.); elisa.borsani@unibs.it (E.B.); 2 Interdepartmental University Center of Research “Adaption and Regeneration of Tissues and Organs (ARTO)”, University of Brescia, 25123 Brescia, Italy

**Keywords:** autism, hippocampus, cornus ammonis, melatonin, oxidative stress, neuroinflammation

## Abstract

Autism spectrum disorder (ASD) is a neurodevelopmental disorder identified by impairments in common social interactions and repetitive behaviors. In ASD patients, substantial morphological alterations have been observed in the hippocampus, which represents an important region for the development of social skills. Melatonin, commonly found in many foods and plants, is also produced by the pineal gland. This indolamine, known to regulate the circadian rhythm, shows antioxidant and anti-inflammatory properties. We therefore hypothesized that melatonin may reduce oxidative stress and inflammation in the hippocampus of ASD patients. We explored our hypothesis using the BTBR mouse, a well-regarded murine transgenic model for ASD. Immediately after weaning, male BTBR and C57BL/6 mice underwent an 8-week treatment with melatonin or vehicle. Later, through immunohistochemistry and the immunoblotting analysis of the hippocampus, we evaluated the overall expression and cellular localization of Nrf2 and SOD1, two enzymes involved in the oxidative stress response. Similarly, we evaluated NLRP3 and NFkB, two mediators of inflammation, and GAD67, an enzyme responsible for the synthesis of GABA. Ultimately, we addressed melatonin’s potential to regulate iron metabolism through a DAB-enhanced Perls reaction assay. Results showed melatonin’s potential for modulating the analyzed markers in BTBR mice, suggesting a potential neuroprotective effect in ASD patients.

## 1. Introduction

Melatonin is an indolamine endogenously produced in the human pineal gland following the light/dark cycle. Through the years, melatonin has been found in a wide range of foods and plants [1], although in an amount that varies significantly among species and varieties and depends on a plethora of other variable factors [2]. Melatonin supplementation has therefore been employed as a valuable sleep-inducing strategy in a plethora of sleep problems such as insomnia and to re-synchronize the circadian rhythm during jetlag and night or shift work [3]. Moreover, extensive research has recognized the antioxidant, anti-inflammatory, and neuroprotective properties of melatonin in a variety of malignancies, in metabolic, neurodegenerative, and neurodevelopmental diseases [4,5,6,7,8]. Among these, autism spectrum disorder (ASD) refers to a wide range of neurodevelopmental disorders sharing two core symptoms: the engagement in restricted and repetitive behaviors and impairments in social interactions and communication [9]. In many patients, these core symptoms are associated with a plethora of co-morbidities such as attention deficiency hyperactivity disorder, intellectual or learning disability, anxiety, depression, obesity, various gastro-intestinal conditions, and sleep disorders [10]. For the latter, melatonin supplementation has been widely employed in ASD patients with positive effect on sleep quality [11] and social scores [12]. Although the etiology of ASD is not yet fully elucidated, fetal neuroinflammation and elevated oxidative stress have been recognized to affect key steps of neuronal development such as synaptogenesis and synaptic pruning [13], ultimately contributing to the onset of ASD symptoms. Accordingly, many studies on various cohorts of ASD patients, when compared to neurotypical individuals, have recorded higher systemic levels in inflammatory [14,15] and oxidative stress markers [16,17]. Another important element of ASD etiology is the altered balance between excitatory and inhibitory synaptic signaling (E/I imbalance). In ASD patients, E/I imbalance has been reported in many central nervous system (CNS) regions such as the prefrontal cortex, amygdala, and hippocampus, likely arising from an alteration in GABAergic neurotransmission [18]. Similar findings regarding the E/I imbalance have also been reported in many genetic and idiopathic murine models of ASD [19,20]. Moreover, anatomical and morphological alterations have been reported in various regions of the CNS of ASD patients [21,22]. Among these, the hippocampus has recently emerged as a key regulatory region for the development of social memories and interindividual bonds [23,24]. Specifically, various murine strains with behavioral patterns similar to ASD core symptoms show hippocampal inflammation [25], reduced synaptic plasticity, and alteration in connectivity in the trisynaptic circuit [26]. In particular, the cornus ammonis 3 (CA3) contains the mossy fibers as non-myelinated axons of the granule cells; these fibers have a central role in intra-hippocampal connectivity and may show a GABAergic phenotype [27]. Moreover, impairing intra-hippocampal connectivity in this specific hippocampal region led to altered social behavior [28]. Interestingly, melatonin deficiency by pinealectomy was reported to affect hippocampal functionality in mice, while exogenous melatonin supplementation after pinealectomy was shown to be protective in hippocampus-related cognitive abilities, suggesting a close link between melatonin and hippocampus development and function [29]. Considering all the above, our study aims to evaluate the neuroprotective, anti-inflammatory, and antioxidant effects of long-term melatonin supplementation on ASD-linked inflammation and oxidative stress in the CA3 region of a widely recognized animal model of idiopathic ASD, the BTBR T^+^Itpr3^tf^/J (BTBR) male mouse strain. Indeed, BTBR mice, especially male individuals, display cognitive and social impairments similar to those found in ASD patients [30], allowing for a precise evaluation of the selected treatment impact on ASD-linked behavioral symptoms. Moreover, the BTBR mouse strain shares with most ASD patients an enhanced oxidative stress and inflammatory state in both peripheral tissues [31,32] and the CNS [33,34]. Finally, morphological defects such as the agenesis of the corpus callosum [35], the excessive growth of the hippocampal pyramidal neurons [36], and long-range connectivity impairments [37] have been previously reported in the BTBR mice strain. To address the oxidative status of BTBR mice neurons in the CA3 region of the hippocampus, we analyzed nuclear factor erythroid 2-related factor 2 (Nrf2) and superoxide dismutase 1 (SOD1) expression, measuring, for both, a significant downregulation.

Nrf2 is a key modulator of oxidative stress response in different cancerous and non-cancerous diseases [38,39]. This is a transcription factor that binds to antioxidant response elements (AREs). Moreover, it has been shown that the Nrf2/Kelch-like ECH-associated protein 1 (Keap1) complex is retained in the cytoplasm. This conformation is susceptible to Nrf2 ubiquitination and degradation under basal conditions. Oxidative stress induces reactive oxygen species (ROS) binding to Keap1, causing a conformational change in Keap1 that inhibits Nrf2 ubiquitination and allows its translocation into the nucleus. In the nucleus, Nrf2 binds to the ARE regions found in the promoter of various antioxidant enzymes, including SOD1, activating their gene transcription. We also found an increase in ferric iron, inflammatory markers such as nuclear factor-kB (NFkB) and NLR family pyrin domain-containing 3 (NLRP3) inflammasome, and a protein needed for Gamma-aminobutyric acid (GABA) synthesis defined as glutamic acid decarboxylase (GAD67). Melatonin treatment proved to significantly improve oxidative stress, inflammation, and synaptic plasticity for all the markers considered.

## 2. Materials and Methods

### 2.1. Animal Treatment

A total of 20 male BTBR T^+^Itpr3^tf^/J mice (JAX™ Mice Strain—IMSR Cat# JAX:002282; Charles River Laboratories Italia S.r.l., Milan, Italy) and 20 male C57BL6/J mice (as control strain) were employed for the experiments (JAX™ Mice Strain—IMSR Cat# JAX: 000664; Charles River Laboratories Italia S.r.l., Milan, Italy). Starting from 3rd week of life the animals were housed in 2 or 3 animals per cage with food and water ad libitum at a constant temperature of 20 °C with a 12 h alternating light–dark cycle. For the first week mice were left housed in the animal facility for acclimatization. All the experimental procedures were approved by the Italian Ministry of Health (N° 446/2018-PR) and followed the National Institutes of Health guide for the care and use of Laboratory animals (NIH publications N°. 8023, revised 1978). The animals of each strain were divided randomly, by an operator unaware of the experimental design, in two groups: 10 animals daily treated with melatonin at a dosage of 10 mg/Kg per oral solution (OS) (gavage), and 10 animals daily treated with a vehicle of melatonin per OS (gavage). Melatonin purchased from Flamma S.p.A. (Bergamo, Italy) was dissolved in ethanol and subsequently in NaCl 75 mM to obtain a final concentration of ethanol of about 1% [40]. Melatonin or vehicle solution were administered daily near the dark period through gavage (100 μL) starting at the 6th week of life for 8 weeks until the end of the 13th week. The animals were trained for starting at the end of the 4th week of life using NaCl 75 mM solution. After treatment, each animal (experimental unit) was behaviorally tested. Subsequently, five mice per group were deeply anesthetized (isoflurane 5%) and transcardially perfused with saline solution followed by 50 mL of 4% paraformaldehyde in phosphate-buffered saline (PBS 0.1 M pH 7.4) for immunohistochemical evaluation. After fixation, brains were carefully removed. The remaining five animals in each group, after sedation, were euthanized by cervical dislocation and their brains were carefully removed, placed in ice, frozen at −20 °C, and subsequently stored at −80°.

### 2.2. Morphological Analysis

The removed brains were postfixed in 4% paraformaldehyde (PBS) for 12 h before being embedded in paraffin using an automatic processor Donatello series 2 (Diapath S.p.A., Bergamo, Italy), cut in sections (7 µm thick) with a semiautomatic microtome Galileo semi-series 2 (Diapath S.p.A., Bergamo, Italy), and stained with hematoxylin–eosin stain (automatic stainer Giotto; Diapath S.p.A., Bergamo, Italy) for general morphology. Subsequently, the stained sections were dehydrated and mounted with DPX (DPX mountant for histology, Sigma, St. Louis, MO, USA) for light microscopy visualization.

### 2.3. Immunohistochemistry

Deparaffinized and rehydrated hippocampal sections were initially incubated at room temperature (RT) for 20 min in 3% H_2_O_2_, then in a 2% goat serum solution (Goat normal serum, Dako Denmark A/S, Glostrup, Denmark) at RT for 60 min, and then in a primary antibody (4 °C overnight) (Table 1). After incubation in the primary antiserum, the sections were sequentially incubated with appropriated biotinylated secondary antibodies (Table 1) and avidin-biotin peroxidase complex (ABC) (Vector Laboratories, Burlingame, CA, USA). The reaction product was revealed using a H_2_O_2_ and diaminobenzidine (DAB) (Sigma, St. Louis, MO, USA) solution as chromogen. To properly visualize the resulting immunopositivity, the sections were counterstained either with Carazzi’s Hematoxylin (Bio-Optica, Milano, Italy) or Gill’s Hematoxylin (Bio-Optica) and subsequently dehydrated and mounted with DPX for light microscopy detection. Immunopositivity was quantified in each by integrated optical density and normalized for the area of the section considered (IOD/Area) with ImagePro premier 9.3 software (2018, Media Cybernetics, Rockville, MD, USA).

### 2.4. DAB-Enhanced Perls Reaction Assay

Hippocampal non-heme iron was evaluated by a DAB-enhanced Perls reaction. The Perls reaction was carried out on deparaffined and rehydrated hippocampal sections using the Perls–van Gieson kit for special staining (Diapath) following the kit instructions. Perls staining enhancement with DAB H_2_O_2_ solution was performed after 20 min of incubation in a 0.01 M NaN_3_ 0.03% H_2_O_2_ solution as previously described by Meguro et al. [41]. The sections were washed briefly in distilled H_2_O in between each step. Sections were then counterstained with Harris’ Hematoxylin (Bio-Optica) and subsequently dehydrated and mounted with DPX for light microscopy detection. The reaction product was quantified by integrated optical density and normalized for the area of the section considered (IOD/Area) with ImagePro premier 9.3 software.

### 2.5. Western Blot

Mice hippocampi from each group were homogenized by mechanical lysis in RIPA buffer (NaCl 150 mM, Tris 50 mM, NP-40 1%, sodium deoxycholate 0.5%, SDS 0.1%, SIGMA) supplemented with protease (cOmplete mini, Roche diagnostic GmbH, Penzberg, Germany) and phosphatase (phosphatase inhibitor cocktail, Sigma) inhibitors. The homogenate was then centrifuged at 17,000× *g* for 20 min at 4 °C, and the supernatant was used for Western blotting assay after protein quantification via the Pierce BCA protein assay kit (Thermo Fisher Scientific, Whaltam, MA, USA). A quantity of 25 µg of each hippocampal lysate was loaded in a 12% polyacrylamide gel and later transferred to a nitrocellulose membrane. After 1 h of blocking in 5% milk (ROTH Gmbh, Karlsruhe, Germany, Art. No. T145.1) tris-buffered saline (TBS) solution, relative protein expression was evaluated through overnight incubation at 4 °C with a primary antibody (Table 2). Primary antibody binding was detected through 1 h incubation at RT with biotinylated secondary antibody (Table 2) or with fluorescent secondary antibodies (Table 2). The membrane was subjected to three washing steps, each lasting 10 min with 0.1% TWEEN 20 TBS solution between each incubation step. The fluorescent signal was directly detected with an Odyssey XF Imaging System (LI-COR, bioscience, Lincoln, NE, USA) and, subsequently, represented as black bands on a white background using ImageJ V 1.8.0 (1.54f software). Band intensity was quantified using the same software, and relative protein expression was calculated by normalizing each protein/actin ratio with the control group (vehicle-treated C57BL/6).

### 2.6. Statistical Analysis

All statistical analyses were performed using R studio (R version 4.2.0). Analysis of variance (two-way ANOVA) was performed followed by Bonferroni’s correction method for multiple comparisons to identify differences considering both strains (C57BL6/J and BTBR T^+^Itpr3^tf^/J) and treatments (vehicle and melatonin). Statistical significance was considered at *p*-value < 0.05.

## 3. Results

### 3.1. Hippocampus General Morphology

**Figure 1:**
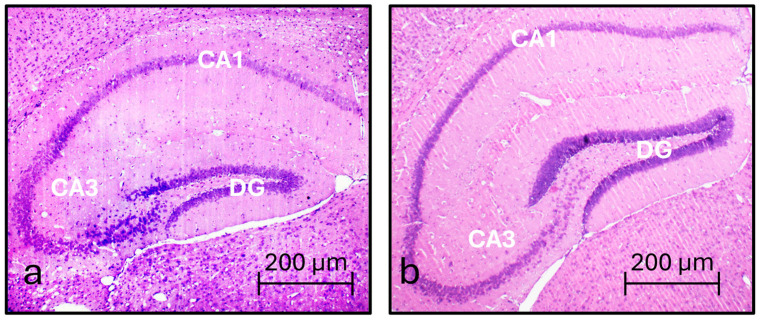
Morphological characterization of the BTBR and C57BL/6 hippocampus. Micrograph (40× magnification) of two coronal brain sections of the hippocampus of BTBR (**a**) and C57BL/6 (**b**) hippocampus. Hippocampal regions are highlighted: cornus ammonis 1 (CA1), cornus ammonis 3 (CA3), dentate gyrus (DG).

The hematoxylin and eosin staining showed, in BTBR mice, an evident decrease in the hippocampal dimension with respect to the C57BL/6 mice (Figure 1). Interestingly, a trend regarding the amount and density of the granule cells forming the dentate gyrus (DG) was visible (Figure 1).

### 3.2. DAB-Enhanced Perls Reaction Assay

**Figure 2:**
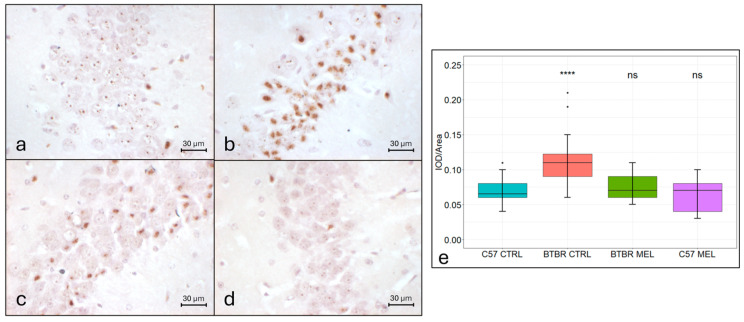
Representative photomicrographs of ferric iron accumulation in the hippocampal CA3 region. DAB-enhanced Perls reaction (N = 4) (magnification 400×) (**a**–**d**). C57BL/6 vehicle-treated group (**a**), BTBR vehicle-treated group (**b**), BTBR melatonin-treated group (**c**), C57BL/6 melatonin-treated group (**d**). Boxplot for the quantitative analysis of DAB-enhanced Perls reaction as IOD/Area (Arbitrary units/μm^2^), **** = *p* < 0.00001, ns = not significant (**e**).

Relative to the vehicle-treated C57BL/6 group (Figure 2a,e; mean ± SEM: 0.0691 ± 0.0021), ferric iron levels in hippocampal neurons were significantly increased (*p* < 0.00001) in the vehicle-treated BTBR group (Figure 2b,e; mean ± SEM: 0.1113 ± 0.0034). In contrast, no significant difference was measured with the melatonin-treated BTBR group (Figure 2c,e; mean ± SEM: 0.074 ± 0.0022; *p* = 0.39) or the melatonin-treated C57BL/6 group (Figure 2d,e; mean ± SEM: 0.065 ± 0.0027; *p* = 0.52). Notably, in all groups, iron was found to be mostly localized in the nucleus of the neurons of the CA3 hippocampal region (Figure 2a–d).

### 3.3. Nrf2

**Figure 3:**
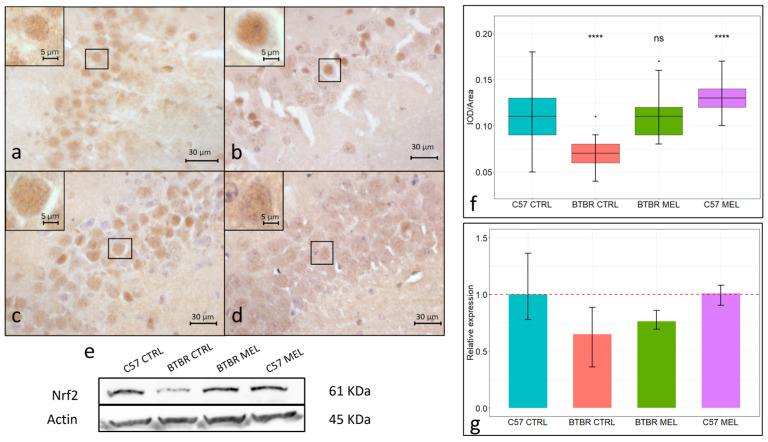
Representative photomicrographs of Nrf2 expression in the hippocampal CA3 region. Immunohistochemical analysis of Nrf2 expression (N = 4) (magnification 400× and insert 1000×) (**a**–**d**). C57BL/6 vehicle-treated group (**a**), BTBR vehicle-treated group (**b**), BTBR melatonin-treated group (**c**), C57BL/6 melatonin-treated group (**d**). Boxplot for the quantitative analysis of Nrf2 expression as IOD/Area (Arbitrary units/μm^2^), **** = *p* < 0.00001, ns = not significant (**f**). Representative Western blot for Nrf2 in hippocampal whole-protein lysate (**e**) and relative expression quantification (N = 3) of Western blot analysis for Nrf2 (**g**).

Nrf2 immunopositivity was found in the hippocampal CA3 region (Figure 3a–e). Nrf2 in the vehicle-treated C57BL/6 group (Figure 3a,e; mean ± SEM: 0.1067 ± 0.0042) was localized both in the cytoplasm and the nucleus, while in the vehicle-treated BTBR group (Figure 3b,e; mean ± SEM: 0.0687 ± 0.002), it was visible mainly in the nucleus and at a significantly lower overall expression level (*p* < 0.00001). Conversely, in the melatonin-treated BTBR group (Figure 3c,e; mean ± SEM: 0.1098 ± 0.0034), Nrf2 was localized in both the cytoplasm and the nucleus while being significantly increased when compared to the vehicle-treated BTBR group (*p* < 0.0001). In the melatonin-treated C57BL/6 group (Figure 3d,e; mean ± SEM: 0.1313 ± 0.0025), a similar Nrf2 subcellular localization and a significant increase in its expression were found when compared to the vehicle-treated C57BL/6 group (*p* < 0.00001). Nrf2 expression in the whole hippocampus was also evaluated through immunoblotting (Figure 3f) and, confirming the trend visible in the immunohistochemical assay, Nrf2 expression was lower in the vehicle-treated BTBR group than in the vehicle-treated C57BL/6 group. Meanwhile, in both BTBR and C57BL/6 mice, the melatonin-treated group showed a higher Nrf2 expression than the respective vehicle-treated group (Figure 3g).

### 3.4. SOD1

**Figure 4:**
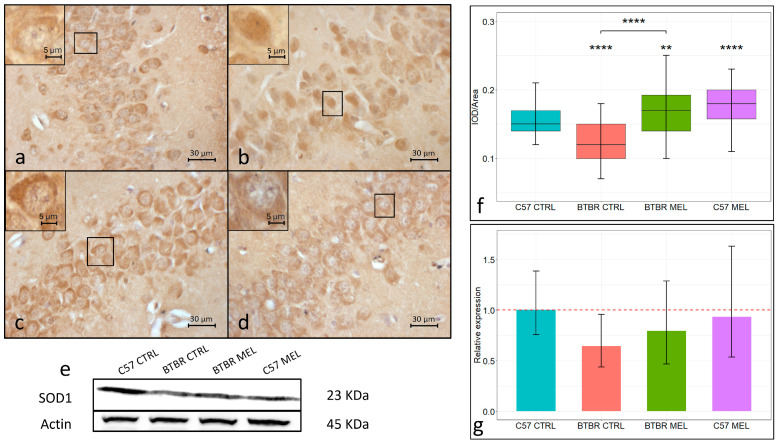
Representative photomicrographs of SOD1 expression in the hippocampal CA3 region. Immunohistochemical analysis of SOD1 expression (N = 4) (magnification 400× and insert 1000×) (**a**–**d**). C57BL/6 vehicle-treated group (**a**), BTBR vehicle-treated group (**b**), BTBR melatonin-treated group (**c**), C57BL/6 melatonin-treated group (**d**). Boxplot for the quantitative analysis of SOD1 expression as IOD/Area (Arbitrary units/μm^2^), ** = *p* < 0.005, **** = *p* < 0.00001 (**f**). Representative Western blot for SOD1 in hippocampal whole-protein lysate (**e**) and relative expression quantification (N = 3) of Western blot analysis for SOD1 (**g**).

SOD1 immunopositivity was also found in the neurons of the hippocampal CA3 region (Figure 4a–e). In the vehicle-treated C57BL/6 group (Figure 4a,e; mean ± SEM: 0.1558 ± 0.024), SOD1 localized almost exclusively in the cytoplasm of neurons, while in the vehicle-treated BTBR group (Figure 4b,e), it showed a diffused localization in both nucleus and cytoplasm paired with an overall decreased expression level (mean ± SEM: 0.1231 ± 0.0036; *p* < 0.0001). In contrast, in the melatonin-treated BTBR group (Figure 4c,e), SOD1 expression was limited to the cytoplasm, and expression levels (mean ± SEM: 0.1707 ± 0.004) were significantly higher than those found in the vehicle-treated BTBR group (*p*< 0.00001). Similarly, SOD1 in the melatonin-treated C57BL/6 group (Figure 4d,e; mean ± SEM: 0.1773 ± 0.0039) localized in the cytoplasm and showed expression levels significantly higher than the vehicle C57BL/6 (*p* < 0.00001). Western blot results for SOD1 (Figure 4f) confirmed the immunohistochemistry trend by showing a diminished expression level in vehicle-treated BTBR mice relative to the vehicle-treated C57BL/6 group. Moreover, in the melatonin-treated BTBR group, SOD1 expression was found to be increased relative to the corresponding vehicle-treated group (Figure 4g).

### 3.5. GAD67

**Figure 5:**
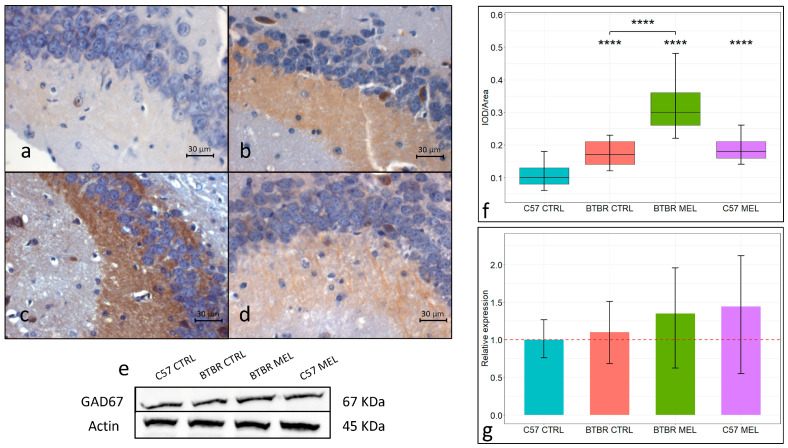
Representative photomicrographs of GAD67 expression in the hippocampal CA3 region. Immunohistochemical analysis of GAD67 expression (N = 4) (magnification 400×) (**a**–**d**). C57BL/6 vehicle-treated group (**a**), BTBR vehicle-treated group (**b**), BTBR melatonin-treated group (**c**), C57BL/6 melatonin-treated group (**d**). Boxplot for the quantitative analysis of GAD67 expression as IOD/Area (Arbitrary units/μm^2^), **** = *p* < 0.00001 (**f**). Representative Western blot for GAD67 in hippocampal whole-protein lysate (**e**) and relative expression quantification (N = 3) of Western blot analysis for GAD67 (**g**).

In all groups, GAD67 immunoreactivity was localized in the mossy fibers found in the CA3 region of the hippocampus (Figure 5a–e). The vehicle-treated C57BL/6 group (Figure 5a,e; mean ± SEM: 0.1095 ± 0.0048) showed a significantly lower (*p* < 0.00001) GAD67 expression relative to the vehicle BTBR-treated mice group (Figure 5b,e; mean ± SEM: 0.1724 ± 0.0055). Similarly, the melatonin-treated BTBR group (Figure 5c,e; mean ± SEM: 0.3126 ± 0.0048) showed a significantly increased GAD67 expression compared to both the C57BL/6 group (*p* < 0.00001) and the vehicle-treated BTBR group (*p* < 0.00001). Comparably, the melatonin-treated C57BL/6 group (Figure 5d,e; mean ± SEM: 0.1847 ± 0.0098) also showed a significant increase in GAD67 level compared with the vehicle-treated C57BL/6 group (*p* < 0.00001). GAD67 expression was also measured in the whole hippocampus through immunoblotting (Figure 5f). Here, with a trend that confirms the immunohistochemical analysis, an increase was found in GAD67 relative expression in the vehicle-treated BTBR group relative to the similarly treated C57BL/6 group. Likewise, both melatonin-treated groups showed a further increase in GAD67 relative expression relative to the corresponding vehicle-treated groups (Figure 5g).

### 3.6. Immunohistochemistry for Inflammation Markers: NLP3 and NFkB

**Figure 6:**
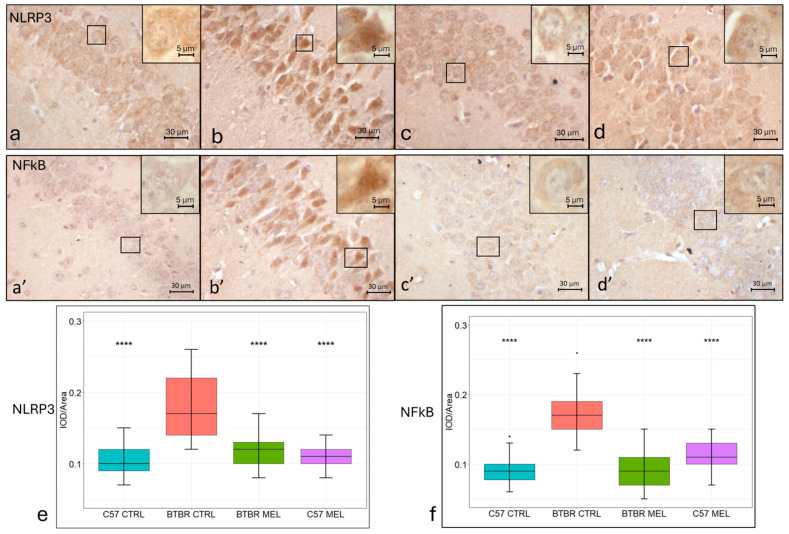
Representative photomicrographs of inflammatory markers’ expression in the hippocampal CA3 region. Immunohistochemical analysis of NLRP3 (N = 4) (**a**–**d**) and of NFKB (N = 4) (**a’**–**d’**) expression (magnification 400× and insert 1000×). C57BL/6 vehicle-treated group (**a**,**a’**), BTBR vehicle-treated group (**b**,**b’**), BTBR melatonin-treated group (**c**,**c’**), C57BL/6 melatonin-treated group (**d**,**d’**). Boxplot for the quantitative analysis of NLRP3 expression as IOD/Area (Arbitrary units/μm^2^), **** = *p* < 0.00001 (**e**). Boxplot for the quantitative analysis of NFkB expression as IOD/Area (Arbitrary units/μm^2^), **** = *p* < 0.00001 (**f**).

Hippocampal NLRP3 immunopositivity was found for every group in the neurons forming the pyramidal layer of the CA3 region (Figure 6a–d). This was in contrast to those in the vehicle-treated C57BL/6 (Figure 6a,e; mean ± SEM: 0.1052 ± 0.0028) group, which showed immunoreactivity for NLRP3 mainly in the cytoplasm. Neurons in the vehicle-treated BTBR group (Figure 6b,e; mean ± SEM: 0.18 ± 0.0052) showed an immunopositivity which was significantly increased (*p* < 0.00001) and equally distributed among the nucleus and cytoplasm. Both the melatonin-treated BTBR (Figure 6c,e; mean ± SEM: 0.1167 ± 0.0027) (*p* < 0.00001) and C57BL/6 groups (Figure 6d,e; mean ± SEM: 0.1107 ± 0.0018) (*p* < 0.00001) showed an NLRP3 cytoplasmic localization and an immunopositivity significantly lower than the vehicle-treated BTBR group. Hippocampal NFkB immunopositivity was found for every group in the neurons of the pyramidal layer of the CA3 region (Figure 6a’–d’). In the vehicle-treated C57BL/6 group (Figure 6a’,f; mean ± SEM: 0.0898 ± 0.0024), NFkB localized in the cytoplasm. NFkB immunopositivity in the vehicle-treated BTBR group (Figure 6b’,f; mean ± SEM: 0.173 ± 0.004) was significantly increased (*p* < 0.0001) when compared to the vehicle-treated C57BL/6 group, and it was localized in both the nucleus and the cytoplasm. In contrast, the melatonin-treated BTBR group (Figure 6c’,f; mean ± SEM: 0.0921 ± 0.0033) showed a significantly lower expression of NFkB relative to the vehicle-treated BTBR group (*p* < 0.0001) and an almost exclusively cytoplasmatic localization for NFkB. Coherently, NFkB immunopositivity in the melatonin-treated C57BL/6 group (Figure 6d’,f; mean ± SEM: 0.1143 ± 0.0022) was found in the cytoplasm and at a significantly lower level relative to the vehicle-treated BTBR group (*p* < 0.0001).

## 4. Discussion

In ASD subjects, hippocampal abnormalities have been reported less consistently from a morphological point of view. Regarding the BTBR animal model, a reduction in dorsal hippocampal volume and hippocampal commissure size has been reported [35,42,43], together with a lateral displacement of the hippocampus and an apparent reduction in the thickness of the hippocampal dentate granule neuron layer, as well as a thinning of the hilus [35] and a reduction in length of the granular layer of the DG [44]. In addition, the two hippocampi (right and left) are excessively separated by tissues which include the roof of the third ventricle and meninges projecting from the falx cerebri [44]. All these observations have been considered and confirmed in our study, together with no specific morphological abnormalities regarding the CA3 area of the hippocampus.

As oxidative stress is now regarded as a key contributor to ASD pathogenesis, in our study, we evaluated gene expression for key regulators of the oxidative stress response in the CA3 region of the hippocampus in all experimental groups. Nrf2 can act as a transcription factor, and it is considered a central regulator of the oxidative stress response. In physiological conditions, Nrf2 is mainly localized in the cytoplasm and associated with Keap1, a protein which inhibits its nuclear localization, while, in response to oxidative stress, it dissociates from Keap1 and can be transferred to the nucleus, where Nrf2 can induce the expression of ARE [45]. A downregulation of Nrf2 has been reported together with an increase in oxidative stress markers in many pathological conditions [46]. Furthermore, a similar downregulation has been recently reported in the hippocampus of BTBR mice [47]. Accordingly, both our immunohistochemical and immunoblotting analyses confirmed an Nrf2 downregulation in the vehicle-treated BTBR CA3 region and in the hippocampus as a whole. Beyond Nrf2 expression, our immunohistochemical assay allows for the analysis of the subcellular localization of Nrf2 in C57BL/6 and BTBR mice in response to melatonin treatment. Specifically, we were able to determine, in the vehicle-treated BTBR group, a higher nuclear localization paired with a lower overall expression of Nrf2, suggesting an increased oxidative stress compared with the C57BL/6 mice. Our data showed a positive effect of melatonin treatment regarding Nrf2 expression in BTBR and C57BL/6 mice in both immunohistochemical and immunoblotting assay; this was also paired with the return to a subcellular localization, like that of the control groups. These data corroborate an extensive amount of previous research, carried out on a plethora of tissues and models, which have individuated melatonin as an activator of the Nrf2-ARE pathway [48]. Among the many Nrf2 targets, SOD1 has been recognized as a valuable contributor to the antioxidant response. SOD1 is an enzyme which catalyzes the dismutation of superoxide radicals into less cytotoxic molecular oxygen and hydrogen peroxide in the cytoplasm. Previous research in different eukaryote models reported that SOD1, in response to elevated ROS levels, can also act as a transcription factor in the nucleus, inducing the expression of many genes involved in DNA damage repair, hydrogen peroxide clearing, andiron and copper metabolism [49]. Our immunohistochemical data showed a significantly lower SOD1 concentration in the hippocampal CA3 region of BTBR mice relative to the control strain, and this trend was consistent with our Western blot analysis in the whole hippocampal lysate. Altered SOD1 activity, due to reduced expression or post-translational modifications, is well known to be impactful on vascular and neuronal health [50,51]. Ultimately, altered SOD1 activity can emerge as consequence of mitochondrial dysfunction in the plasma of ASD patients [52], although SOD1 downregulation has been reported both to be positively correlated [53] and to not be correlated with ASD symptoms’ severity [54]. Interestingly, in the hippocampal neurons of vehicle-treated BTBR mice, SOD1 localized in both nucleus and cytoplasm; in contrast, melatonin restored the cytoplasmic localization typical of both C57BL/6 groups. Altogether, these data hint at a higher oxidative stress in the hippocampus of BTBR mice relative to the control strain, which can impact Nrf2 and SOD1 subcellular localization, potentially lowering BTBR mice’s response to ROS production. Moreover, 8-week melatonin treatment significantly reduced neuronal oxidative stress in the BTBR hippocampus, restoring the physiological Nrf2 and SOD1 concentrations and subcellular localization. Our data showed, as a potential consequence of increased oxidative stress, an increased deposition in BTBR mice of ferric iron in hippocampal neurons, possibly leading to ferroptosis. Ferroptosis is a newly recognized type of cell death caused by the accumulation of intracellular iron, which promotes lipid peroxidation. This cell death is now recognized as an important contributor to CNS injury and neurodegenerative diseases. Recent research suggests that the accumulation of reactive oxygen species and neuroinflammation are key contributors in triggering ferroptosis. The latter was suggested to be involved in hippocampal neuron damage and loss in murine models subjected to chronic cerebral hypoperfusion [55,56]. Many studies suggested that inhibiting the production of ROS could significantly reduce the occurrence of ferroptosis. Accordingly, some data have shown that ROS inhibitors (such as vitamin E and melatonin) can effectively inhibit ferroptosis. Although ferroptosis inhibitors are widely used in a variety of neurological diseases, the use of ferroptosis inhibitors differs following the varying disease etiologies [57,58]. Our results confirmed the action of melatonin as an antioxidant by reducing iron deposits in the hippocampal neurons of BTBR mice. Taken together, these data pave the way to further in vivo and clinical experimentations exploring the connections between oxidative stress, iron metabolism, and neurodevelopment.

We also evaluated GAD67, a pyridoxal phosphate dependent decarboxylase, which catalyzes the synthesis of GABA, the main inhibitory neurotransmitter. In the hippocampus, GAD67 mainly localizes in the DG granule cells’ axons connecting to the CA3 pyramidal neurons’ dendrites. Our immunohistochemical data showed a significant increase in GAD67 expression in the mossy fibers in the BTBR strain relative to the C57BL/6 strain, and this was confirmed by our Western blot analysis on the whole hippocampus. A similar upregulation of GAD67 in the hippocampus has been reported before in murine models for fetal nicotine exposure [59] and fetal alcohol exposure [60]. Increased GAD67 immunopositive cells were also reported following elevated astrocytes proliferation during development, and this was paired with ASD-like behaviors and enhanced inhibitory transmission in the hippocampus [61]. Our data, in accordance with the reported literature and well-regarded hypothesized ASD etiology, hint at a significant role of E/I imbalance, together with elevated oxidative stress and inflammation, in ASD morphological alterations and behavioral symptoms. Our data also showed how melatonin treatment elevated GAD67 expression level in the CA3 and mossy fibers of both mice strain considered. In the hippocampal CA3 region, the over-expression of GAD67 through viral transduction was also reported to be a protective factor against temporal lobe epilepsy in a murine model [62]. Together, our data strengthen the E/I hypothesis of ASD etiology and, subsequently, suggest a potential role for long-term melatonin treatment as a regulator of E/I balance in the hippocampus. Furthermore, the potential for nutraceuticals supplementation could be addressed, also in human studies, as a new and complementary tool for neurotransmitter regulation.

Our data report a significant increase in overall NFkB expression in hippocampal CA3 neurons, which was then normalized by long-term melatonin treatment. Notably, the increased NFkB nuclear localization visible in the untreated BTBR group was ameliorated by long-term melatonin supplementation, suggesting a lower inflammatory profile and a lower induction of inflammatory molecules following the treatment. Specifically, NFkB is a central regulator of inflammatory response [63,64], and it is retained in the cytoplasm when inactivated by its endogenous inhibitor IkB, which prevents nuclear translocation [65]. NFkB is activated, through phosphorylation, by a plethora of factors, including ROS, cytokines, and other signals such as neurotransmitters and nerve growth factors. This results in an increased NFkB resistance to degradation, its translocation in the nucleus [66], and the transcription of various pro-inflammatory genes, including cytokines, chemokines, and inflammasome complex elements such as NLRP3 [67]. Notably, an interaction between the NFkB pathway and the Nrf2/ARE pathway has been previously reported. Indeed, free Keap1 prevents the degradation of IkB, thereby inhibiting the NFkB pathway, and activated NFkB also inhibits Keap1 from interfering with the nuclear transcription of Nrf2 [68]. Following the broad range of stimuli that were documented to activate neuronal NFkB, among which are neural growth factor, brain-derived neurotrophic factor, and excitatory neurotransmitters [69], the NFkB pathway has been implicated in mice in the regulation of cognitive behaviors, learning, and memory [70]. Collectively, these effects are consistent with a role for NFkB in enhancing excitatory synaptic function. In BTBR mice, peripheral inflammatory mediators activate transcription factors such as NFkB in peripheral immune cells [71], likely worsening systemic inflammation. Our data, besides characterizing the inflammatory status of CA3 hippocampal neurons, confirm the well-known anti-inflammatory properties of melatonin reported in previous studies on a plethora of diseases and models. NFkB is known for its role as a central mediator of the priming signal of NLRP3 inflammasome activation that is exerted by inducing the transcriptional expression of NLRP3 [67]. The NLRP3 inflammasome is pivotal in the processing of active caspase-1 and the downstream maturation of IL-1β [72]. These effects further result in the recruitment of inflammatory cells into the brain, amplifying the inflammatory reaction. Our work focused on NLRP3 since it is “apical” in the inflammation cascade and a potential target for natural compounds known for their anti-inflammatory effect, among which is melatonin. Our results in the BTBR mouse strain showed that ASD may be linked to an increase in NLRP3 in hippocampal neurons. These results fit with the study by Ventura and colleagues demonstrating the NLRP3 inflammasome’s involvement in schizophrenia-like behavior in young animals exposed to maternal immune activation. In this study, the authors postulated that the inflammatory insult during central nervous system development may contribute to long-term changes in the brain that may persist into adulthood [73]. Moreover, our results agree with a previous study which showed that melatonin attenuates Lipopolysaccharide-induced acute depression-like behavior and NLRP3 inflammasome activity in microglia via the Sirtuin 1/Nrf2 pathway [74]. The activation of the NLRP3 inflammasome may be responsible for maintaining a persistent neuroinflammatory state in the CNS, which may be associated with behavioral or mood disorders and neurodegeneration diseases. On this matter, long-term treatment with MCC950, a highly selective inhibitor of NLRP3, was shown to be beneficial in the treatment of mood disorders [75], neurodevelopmental diseases [76], and neurodegenerative diseases such as Alzheimer’s and Huntington’s disease [77]. Accordingly, we showed that long-term melatonin supplementation can exert similar anti-inflammatory and neuroprotective effects, probably by acting on the Nrf2-NFkB-NLRP3 pathway. Taken together, our data confirm the anti-inflammatory action of melatonin in the hippocampus and, therefore, suggest a potential role in the treatment of ASD symptoms. Further studies could explore cell-specific responses to inflammatory signals, their impact during neurodevelopment, and effective tools to regulate them.

## 5. Conclusions

We previously demonstrated in the prefrontal cortex of the same murine model that melatonin supplementation, starting after weaning, reduces the redox imbalance and promotes synaptic plasticity. The results of this study confirmed the involvement of oxidative stress and inflammation in ASD. Moreover, they shed new light on the potential use of melatonin as a hippocampal neuron protector in these pathologies. This is achieved by alleviating oxidative stress and inflammation through different pathways, suggesting a potential role for melatonin as an adjuvant therapy in ASD treatment. However, additional in vitro and in vivo studies should be conducted to better investigate the mechanisms underlying the beneficial effects of melatonin supplementation on ASD patients, especially considering the heterogeneous manifestations and the complex etiology of autism.

## Figures and Tables

**Table 1:** List of primary and secondary antibodies used for immunohistochemical assays.

Antibody	Dilution	Distributor, Cat. Num.
anti-SOD1 mouse monoclonal antibody	1:300	Santa Cruz Biotechnology, Santa Cruz, CA, USA, sc-101523
anti-Nrf2 rabbit polyclonal antibody	1:300	Abcam, Cambridge, UK, ab31163
anti-GAD67 mouse monoclonal antibody	1:300	Abcam, MAB5406
anti-NFkB p65 rabbit polyclonal antibody	1:200	Abcam, ab16502
anti-NLRP3 mouse polyclonal antibody	1.200	Abcam, ab214185
polyclonal goat anti-rabbit biotinylated antibody	1:50	DakoCytomation, Glostrup, Denmark E0432
polyclonal goat anti-mouse biotinylated antibody	1:50	DakoCytomation, E0433

**Table 2:** List of primary and secondary antibodies used for Western blot assays.

Antibody	Dilution	Distributor, Cat. Num.
anti-SOD1 mouse monoclonal antibody	1:1000	Santa Cruz Biotechnology, sc-101523
anti-Nrf2 rabbit polyclonal antibody	1:1000	Abcam, ab31163
anti-GAD67 mouse monoclonal antibody	1:2000	Abcam, MAB5406
monoclonal anti-β-Actin antibody (mouse)	1.2500	Sigma, A5441
IRDye^®^ 800CW Goat anti-Rabbit IgG Secondary Antibody	1:2500	LI-COR bioscience, 926-32211
IRDye^®^ 680RD Goat anti-Mouse IgG Secondary Antibody	1.2500	LI-COR bioscience, 925-68070
polyclonal goat anti-rabbit biotinylated antibody	1:2500	DakoCytomation, E0432
polyclonal goat anti-mouse biotinylated antibody	1:2500	DakoCytomation, E0433

## Data Availability

The data underlying this article will be shared upon reasonable request to the corresponding author.
